# Clinical relevance of expanded quantitative urine culture in health and disease

**DOI:** 10.3389/fcimb.2023.1210161

**Published:** 2023-08-01

**Authors:** Nadia S. Deen, Akash Ahmed, Nazifa Tabassum Tasnim, Nabila Khan

**Affiliations:** Microbiology Program, Department of Mathematics and Natural Sciences, BRAC University, Dhaka, Bangladesh

**Keywords:** expanded quantitative urine culture (EQUC), enhanced urine culture, urinary tract infection (UTI), urgency urinary incontinence (UUI), microbiome, urobiome, urine, bladder

## Abstract

“Expanded quantitative urine culture (EQUC)” is an enhanced culture protocol for the detection of viable microbes in urine specimens. Using a large volume of urine and different sets of cultural conditions, EQUC is able to uncover a wide range of bacteria and fungi (yeasts) that were otherwise undetected by the standard urinary culture. In addition to common urinary pathogens, EQUC has been shown to detect emerging and new pathogens, and commensal microbiota. Although the usefulness of EQUC protocol in clinical set up has not yet been fully established, recent studies have demonstrated that EQUC can provide valuable information regarding symptom resolution, treatment responses and diagnosis of major urinary disorders including urinary tract infections, urinary incontinence and other lower urinary tract symptoms. EQUC may also help in evaluating the utility of beneficial microbiota as biotherapeutics. This narrative minireview describes the current research findings regarding the clinical utility of EQUC in characterizing the role of urinary microbiome and uropathogens in health and disease. The literature which are written in English, available on “PubMed” and contain any of the terms- “expanded quantitative urine culture”, “enhanced quantitative urine culture” and “EQUC” in the abstracts were used as the source articles to prepare this minireview.

## 1 Introduction

It was not long ago when the human bladder was believed to be sterile (Wolfe and Brubaker, 2015; Thomas-White et al., 2016). Using 16s ribosomal RNA (rRNA) sequencing, Wolfe and colleagues for the first time described that the bladder of healthy females contained uncultivable bacteria (Wolfe et al., 2012). However, they were unable to detect whether those uncultivable bacteria were viable. Two years later, using the protocol “expanded quantitative urine culture (EQUC)”, also known as “enhanced quantitative urine culture”, they showed that female urine harbored communities of live bacteria (Hilt et al., 2014). Since then, EQUC, in addition to or independent of standard urine culture (SUC) and next generation sequencing (NGS), have been used to detect urinary microbiota/microbiome (urobiome) from healthy and diseased individuals.

EQUC offers several advantages relative to SUC and NGS. Since the first description of EQUC in 2014, the protocol has been updated in a number of ways to utilize a broad range of growth media, culture conditions and incubation time to detect bacteria and some fungi, particularly yeasts, in urine samples (**Table 1**). EQUC is able to detect microbes in urine samples that are considered as “no growth” by SUC (Hilt et al., 2014; Brubaker and Wolfe, 2016). One of the strengths of EQUC is its ability to detect slow-growing anaerobic, microaerophilic and fastidious bacteria, in addition to aerobic and facultative anaerobes (Southworth et al., 2019). EQUC is also capable of detecting atypical and subthreshold bacterial species at a detection level of as low as 10 colony-forming unit (CFU)/ml (Xu et al., 2021). Since EQUC detects viable microbes, it is possible to evaluate the antibiotic sensitivity profiles of the bacteria analyzed by EQUC (Thapaliya et al., 2020; Xu et al., 2021). Without whole genome sequencing, NGS alone cannot provide data on antibiotic resistance.

**Table 1:** Detection of urotypes by SUC vs. EQUC.

Reference	Objectives of the study	Protocol	Detection level of bacteria	% of culture positive sample	Number of urotype detected
(Hilt et al., 2014)	Detection of cultivable bacteria in adult females with or without overactive bladder (OAB).	SUC: 0.001 ml of urine onto 5% sheep BAP and MacConkey agars aerobically at 35°C for 24 hours. EQUC: *For aerobic fastidious bacteria:* 0.1 ml of urine onto BAP, chocolate agar and CNA in 5% CO_2_ at 35°C for 48 hours. *For aerobic bacteria:* 0.1* ml* of urine onto a second set of BAPs at room atmosphere at 35°C and 30°C for 48 hours. *For microaerophilic and anaerobic bacteria:* 0.1 ml of urine onto each of two CDC anaerobe 5% sheep blood agar plates in either a Campy gas mixture or under anaerobic conditions at 35°C for 48 hours. *For any bacterial species that may be present at less than 10 CFU/ml:* 1.0 ml of urine in thioglycolate medium and incubated aerobically at 35°C for 5 days. If growth was visually detected in the thioglycolate medium, the medium was mixed and a few drops were plated on BAP and CDC anaerobe 5% sheep blood agars for isolation and incubated aerobically and anaerobically at 35°C for 48 hours.	SUC: 10^3^ CFU/ml EQUC: 10 CFU/ml	SUC: 6.15% EQUC: 80%	SUC: not specified EQUC: 35 genera and 85 species
(Pearce et al., 2014)	Comparison of female urinary microbiome with and without urgency urinary incontinence (UUI).	SUC and EQUC: Same as described by (Hilt et al., 2014)	SUC: 10^3^ CFU/ml EQUC: 10 CFU/ml	SUC: 7.78% EQUC: 78.9%	SUC: not specified EQUC: 36 genera
(Price et al., 2016)	Evaluation of enhanced techniques to improve detection of clinically relevant microorganisms from females with and without urinary tract infection (UTI).	SUC: same as described by (Hilt et al., 2014) Expanded spectrum EQUC: *For aerobic bacteria:* 0.1, 0.01 and 0.001 ml of urine onto BAP and MacConkey agar at 35°C for 24 and 48 hours. For aerobic fastidious bacteria: 0.1, 0.01 and 0.001 ml of urine onto BAP, chocolate agar and CNA in 5% CO_2_ at 35°C for 24 and 48 hours. *For microaerophilic and anaerobic bacteria:* 0.1, 0.01 and 0.001 ml of urine onto each of two CDC anaerobe 5% sheep blood agar plates in either a Campy gas mixture or under anaerobic conditions at 35°C for 48 hours. Streamlined EQUC: 0.1 ml of urine onto BAP, CNA and MacConkey agars in 5% CO_2_ for 48 hours.	SUC and expanded spectrum EQUC: varied per condition	SUC: 33% Expanded spectrum EQUC: 93% Streamlined EQUC: 84% of expanded spectrum EQUC	SUC: not specified Expanded spectrum EQUC: Non-UTI (75 species), UTI (66 species)
(Thomas-White et al., 2016)	Detection of association of the characteristics of microbiome/microbiota with clinically relevant treatment response to oral UUI medication.	SUC and EQUC: same as described by (Hilt et al., 2014)	SUC: 10^3^ CFU/ml EQUC: 10 CFU/ml	SUC: 7.88% EQUC: 81.3%	SUC: not specified EQUC: control (36 organisms) and UUI (80 organisms) (at the baseline)
(Dune et al., 2017)	Evaluation of urinary symptoms and their associations with UTI in urogynecologic patients.	SUC: same as described by (Hilt et al., 2014) Expanded spectrum EQUC *For aerobic bacteria:* 0.1, 0.01 and 0.001 ml of urine onto BAP and MacConkey agar at 35°C for 24 hours. *For aerobic fastidious bacteria:* 0.1, 0.01 and 0.001 ml of urine onto BAP, chocolate agar and CNA in 5% CO_2_ at 35°C for 24 and 48 hours. *For microaerophilic and anaerobic bacteria*: 0.1, 0.01 and 0.001 ml of urine onto each of two CDC anaerobe 5% sheep blood agar in either a Campy gas mixture or under anaerobic conditions at 35°C for 48 hours.	SUC: 10^3^ CFU/ml EQUC: 10 CFU/mL	SUC: 38% EQUC: 93%	SUC: 11 species EQUC: 36 genera and 98 species
(Jacobs et al., 2017)	Discovery of the maternal bladder microbiota.	SUC: same as described by (Hilt et al., 2014) EQUC: 0.1 ml of urine onto BAP, CNA and chocolate agar in aerobic, anaerobic and CO_2_ at conditions 35°C for 48 hours.	SUC: 10^3^ CFU/ml EQUC: 10 CFU/ml	SUC: no growth EQUC: 66.7%	SUC: no bacteria detected EQUC: 34 genera
(Thomas-White et al., 2018)	Culturing and genome sequencing of female bladder bacteria.	SUC and EQUC: Same as described by (Hilt et al., 2014)	SUC: 10^3^ CFU/ml EQUC: 10 CFU/ml	SUC: not specified EQUC: 66.4%	SUC: not specified EQUC: 36 genera and 78 species
(Bajic et al., 2020)	Evaluation of the male bladder microbiome relating to the lower urinary tract symptoms.	SUC: same as described by (Hilt et al., 2014) EQUC: 0.1 ml of urine onto BAP, CNA, chocolate agar and anaerobic blood agar in aerobic, anaerobic and CO_2_ conditions at 35°C for 48 hours.	SUC: 10^3^ CFU/ml EQUC: 10 CFU/ml	SUC: not specified EQUC: 96% (voided urine specimens) and 29% (catheterized urine specimens)	SUC: not specified EQUC: 37 genera
(Southworth et al., 2019)	Comparison of the standard urine collection to the Peezy midstream device in women.	SUC: not performed EQUC: 0.1mL urine onto BAP, chocolate agar, CNA, CDC anaerobe BAP in 5% CO_2_, aerobic conditions, Campy gas mixture (5% O2, 10% CO2, 85% N) or anaerobic conditions) at 35°C for 48 hours.	SUC: not performed EQUC: 10 CFU/mL	SUC: not performed EQUC: not specified	SUC: not performed EQUC: not specified
Chen et al. (2020)	Comparison of the urethral microbiota with the bladder urinary microbiota in presence of the lower urinary tract symptoms.	SUC: not performed EQUC: 0.1 ml catheterized urine or 0.01 ml voided, periurethral or urethral specimens onto diverse types of media with incubation in diverse environments at 35°C for 48 hours.	SUC: not performed EQUC: 10 CFU/ml (catheterized samples) or 100 CFU/ml (other samples)	SUC: not performed EQUC: not specified	SUC: not performed EQUC: 35 genera
(Price et al., 2020a)	Evaluation of the bladder bacterial diversity in continent and incontinent women.	SUC and EQUC: same as described by (Hilt et al., 2014)	SUC: 10^3^ CFU/ml EQUC: 10 CFU/mL	SUC: no growth EQUC: Control (57%), UUI (81%) and stress UI (86%)	SUC: no bacteria detected EQUC: not specified
(Price et al., 2020b)	Assessment of the temporal dynamics of the adult female lower urinary tract microbiota.	SUC: not performed EQUC: 0.01 ml of urine onto BAP, CNA, and CDC anaerobe 5% BAP in 5% CO_2_ at 35°C for 48 hours or anaerobic conditions 35°C for 48 hours.	SUC: not performed EQUC: not specified	SUC: not performed EQUC: not specified	SUC: not performed EQUC: not specified
(Thapaliya et al., 2020)	Detection of under−diagnosed pediatric UTI.	SUC: same as described by (Hilt et al., 2014) EQUC: 0.1, 0.01 and 0.001 ml of urine onto BAP and chocolate agar plates incubated in 5% CO_2_ at 35°C for 48 hours; BAP and MacConkey agars were incubated aerobically at 35°C for 48 hours.	SUC and EQUC: varied per conditions	SUC: 12.80% EQUC: 16.15%	SUC: 8 species EQUC: 10 species
(Burnett et al., 2021)	Determination of the association of clinical profiles with urobiome composition in women.	SUC and Streamlined EQUC: same as described by (Price et al., 2016)	SUC and Streamlined EQUC: same as described by (Price et al., 2016)	SUC and EQUC: not specified	SUC and EQUC: not specified
(Barnes et al., 2021)	Diagnosis of urinary tract infections in women *via* standard versus expanded cultures.	SUC: same as described by (Hilt et al., 2014) EQUC: 0.1 mL of urine onto BAP, MacConkey agar and CNA in 5% CO2 at 37°C for 48 hours.	SUC: 10^3^ CFU/ml EQUC: 10 CFU/ml	SUC: 63% and EQUC: 74%	SUC: not specified EQUC: 28 genera
(Hrbacek et al., 2021)	Assessment of the urine sampling methods to detect the male urinary microbiota.	EQUC: 0.1 ml of urine onto Columbia blood agar 37°C for 48 hours; CBA incubated at 30°C for 48 hours; CBA incubated at 37°C in a 5% CO_2_ incubator for 48 hours; chocolate agar at 37°C in a 5% CO_2_ incubator for 48 hours; Schaedler blood agar at 37°C in a Campy gas mixture (5% O_2_, 10% CO_2_ and 85% N); thioglycolate broth incubated at 37°C for 5 days, then inoculated on CBA and incubated at 37°C for another 48 hours.	EQUC: 10 CFU/ml	EQUC: 97% (first-catch voided urine), 87% (mid-stream vided urine) sand only 13% (catheterized samples)	EQUC: 13 genera and 23 species
(Jacobs et al., 2021)	Detection of the cultivable bacteria in the urine of women with interstitial cystitis.	SUC: not performed EQUC: same as described by (Hilt et al., 2014; Price et al., 2016)	SUC: not performed EQUC: 10 CFU/mL	SUC: not performed EQUC: Control (40%) and interstitial cystitis (IC)/painful bladder syndrome (PBS) (49%)	SUC: not performed EQUC: Control: 14 species and IC/PBS: 19 species
(Vaughan et al., 2021)	Detection of the urinary microbiome in postmenopausal women with recurrent UTI.	SUC and EQUC: same as described by (Hilt et al., 2014)	SUC: 10^3^ CFU/ml EQUC: 10 CFU/mL	SUC: no growth EQUC: 59.40%	SUC: no bacteria detected EQUC: not specified
(Halverson et al., 2022a)	Assessment of the correlation of symptom improvement with urobiome characteristics in adult women prescribed mirabegron for UUI treatment.	SUC: not performed EQUC: Same as described by (Hilt et al., 2014)	SUC: not performed EQUC: 10 CFU/mL	SUC: not performed EQUC: 90% at baseline	SUC: not performed EQUC: 34 genera
(Halverson et al., 2022b)	Comparison of urobiome changes based on OAB treatment in adult females.	SUC: not performed EQUC: Same as described by (Hilt et al., 2014)	SUC: not performed EQUC: 10 CFU/mL	SUC: not performed EQUC: not specified	SUC: not performed EQUC: not specified
(Hochstedler et al., 2022)	Assessment of the collection and culture methods to detect urinary microbiota of women with recurrent UTIs.	SUC and streamlined EQUC as described by (Price et al., 2016)	SUC and streamlined EQUC as described by (Price et al., 2016)	SUC: Catheterized UTI-associated microbes (39.5%) and voided UTI-associated microbes (57.1%) EQUC: Catheterized UTI-associated microbes (53.5%) and voided UTI-associated microbes (90.5%)	SUC: Catheterized UTI-associated microbes (9) and voided UTI-associated microbes (24) EQUC: Catheterized UTI-associated microbes (24) and voided UTI-associated microbes (56)
(Joyce et al., 2022)	Evaluation of the microbiome of adult women with the lower urinary tract symptoms.	SUC: not performed EQUC: Same as described by (Hilt et al., 2014)	SUC: not performed EQUC: 10 CFU/mL	SUC: not performed EQUC: UTI (94.4%), UUI (92.1%), stress UI (99.5%), IC/PBS (60.2%) and controls (57.5%)	SUC: not performed EQUC: not specified
(Storm et al., 2022)	Evaluation of the pediatric urinary microbiome.	SUC: not performed EQUC: Same as described by (Hilt et al., 2014; Pearce et al., 2014; Price et al., 2016)	SUC: not performed EQUC: Same as described by (Hilt et al., 2014; Pearce et al., 2014; Price et al., 2016)	SUC: not performed EQUC: 60%	SUC: not performed EQUC: not specified
(Vaughan et al., 2022)	Assessment of concordance of urinary microbiota detected by 16S rRNA sequencing vs EQUC.	SUC: not performed EQUC: Same as described by (Hilt et al., 2014)	SUC: not performed EQUC: 10 CFU/mL	SUC: not performed EQUC: not specified	SUC: not performed EQUC: not specified

BAP, Blood Agar Plate; CNA, Columbia Naladixic Acid Agar; CDC, Centers for Disease Control; CFU, Colony Forming Units.

Although EQUC has not been used regularly in a clinical set up due to lack of sufficient data, accumulating evidence have demonstrated the clinical relevance of EQUC in terms of symptoms resolution, treatment response and diagnosis. In the following sections of this review, recent data on the utility of EQUC with respect to several urinary conditions (**Table 2**) will be discussed.

**Table 2:** Comparisons between participants of different urinary tract conditions reported in the EQUC urinary microbiota studies.

Urinary tract condition(s) evaluated	Inclusion criteria for participants	Exclusion criteria for participants	No of participants	Gender of participants	Average age of participants	Reference
Overreactive bladder (OAB)	Patients undergoing OAB treatment (cases) and a comparison group of patients undergoing benign gynaecologic surgery (controls).	Participants with clinical evidence of urinary tract infection (UTI) (i.e., urine culture negative and absence of clinical UTI diagnosis).	41 cases and 24 controls	Females	Not specified	(Hilt et al., 2014)
Urgency urinary incontinence (UUI)	Patients with UUI (cases) and a comparison group of individuals without UUI (controls).	Participants with current UTI or history of recurrent UTI, antibiotic exposure in the past 4 weeks, immunologic deficiency, neurological disease known to affect the lower urinary tract, pelvic malignancy or radiation, untreated symptomatic pelvic organ prolapses (POP) greater than POP-Q stage II (vaginal protrusion more than 1 cm outside of the vaginal hymen) or pregnancy.	60 cases and 58 controls	Females	Cases: 63 years Controls: 49 years	(Pearce et al., 2014)
Urinary tract infection (UTI)-like symptoms	Participants included patients who said yes (cases)/no (controls) to the question “Do you feel you have a UTI?”	Participants with an age of less than 18 years, pregnancy, catheterization (indwelling or intermittent), or insufficient English skills to complete study measures.	75 cases and 75 controls	Females	62.3 years	(Price et al., 2016)
Urgency urinary incontinence (UUI)	Patients seeking UUI treatment (cases) and a cohort of patients with benign gynecologic conditions who underwent surgical procedure (controls).	Same as described by (Pearce et al., 2014)	74 cases and 60 controls	Females	Cases: 61.5 years Controls: 49 years	(Thomas-White et al., 2016)
Urinary tract infection (UTI)	Same as described by (Price et al., 2016)	Same as described by (Price et al., 2016)	75 cases and 75 controls	Females	62.3 years	(Dune et al., 2017)
Asymptomatic bacteriuria (ASB)	Pregnant patients presenting to labour and delivery.	Participants with an age of less than 18 years, insufficient English skills to complete study measures and had used antibiotics within the past 4 weeks.	51	Females	30 years	(Jacobs et al., 2017)
Different undefined urinary tract conditions	Participants were recruited from other studies.	Same as described by (Pearce et al., 2014)	38 (asymptomatic) and 39 (symptomatic)	Females	Not specified	(Thomas-White et al., 2018)
Lower urinary tract symptoms (LUTS)	Patients undergoing benign prostate enlargement (BPE)/lower urinary tract symptoms (BPE/LUTS) surgery (cases) and patients undergoing non-BPE/LUTS surgery (controls).	Participants with preoperative symptomatic UTI, current urolithiasis, antibiotic use within 30 days of sample acquisition, a history of genitourinary malignancy, immunocompromised state, active malignancy, urethral stricture, urinary retention, catheterization, other genitourinary instrumentation (including prostate biopsy, urodynamics, etc.), or recent abdominal/pelvic surgery.	28 cases and 21 controls	Males	61.7 years	(Bajic et al., 2020)
Conditions without any urinary tract disorders	Adult healthy women of 18 years old or older who could speak fluent English, were ambulatory, could view video-based instruction for the collection method, and answered yes to the query “do you feel your bladder is full enough to void.”	Participants with a history of recurrent UTI, POP, UUI or urinary stress incontinence, current use of antibiotics, post-menopausal status, pregnancy, current menstruation or the use of intermittent self-catheterization.	62	Females	28.8 years	(Southworth et al., 2019)
Conditions with or without pelvic floor symptoms	Women of 18 years old or older who presented for evaluation of pelvic floor symptoms.	Participants with UTI symptoms, antibiotic use within 30 days, history of gynecologic malignancy, pelvic radiation, urethral resection, indwelling urinary catheter, use of intermittent self-catheterization or pregnancy.	49	Females	55 years (median age)	Chen et al. (2020)
Urinary incontinence (UI)	Participants included urine incontinent (cases) and urine continent (controls) individuals.	Participants with systemic antibiotic use in prior 4 weeks, current pregnancy, current therapeutic catheterization (indwelling or intermittent) or insufficient English skills to complete study measures.	159 cases [Stress UI (SUI): 50 and UUI: 109] and 150 continent controls	Females	Stress UI: 54, UUI: 61 and continent Controls: 47	(Price et al., 2020a)
Conditions without lower urinary tract symptoms (LUTS)	Participants were non-symptomatic premenopausal.	Participants with current pregnancy, antibiotic or probiotic usage, or a plan to vacation for more than 7 days during the time of specimen collection.	12 cases and 8 controls	Females	29 years	(Price et al., 2020b)
Urinary tract infection (UTI)	Infants and children seeking treatment for presumed UTI.	Participants who Previously known the history of antimicrobial therapy within 48 h before attending the hospital.	570	Females (319) and males (251)	Varied per age group	(Thapaliya et al., 2020)
Recurrent urinary tract infection (rUTI)	Participants meeting diagnostic criteria for rUTI (>3 symptomatic standard culture positive UTIs in one year or 2 in six months) and seeking rUTI care.	Participants with known anatomic abnormalities of the urogenital tract, neurologic or immunologic disease, a history of bladder malignancy, or current systemic infection.	43	Females	67 years	(Burnett et al., 2021)
Urinary tract infection (UTI)	Adult women of 18 years old or older who reported symptoms of a UTI were screened for eligibility.	Participants who were on antibiotics, unable to communicate or read in English, under age 18, pregnant, use of an indwelling urinary catheter, treated empirically on enrolment day, did not have sufficient collected urine volume for analysis, were performing intermittent self-catheterization, or declined to be catheterized.	225	Females	66.6 years	(Barnes et al., 2021)
Benign or malignant conditions of the urinary tract	Participants undergoing endoscopic procedures for benign or malignant conditions of the urinary tract.	Participants with a positive result of standard urine culture preoperatively, presence of foreign body in the urinary bladder and have use of antibiotics for any medical condition in the past 6 weeks.	49	Males	71.3 years (median age)	(Hrbacek et al., 2021)
Interstitial cystitis (IC)/painful bladder syndrome (PBS)	Women ≥18 years old were eligible for the IC/PBS cohort if clinicians believed they fit the following description: “An unpleasant sensation (pain, pressure, discomfort) perceived to be related to the urinary bladder, associated with lower urinary tract symptoms of more than six weeks duration, in the absence of infection or other identifiable causes.” Participants who did not have bladder pain were eligible for the control cohort.	Being currently pregnant, experiencing vaginal itching/burning/discharge or gross hematuria, had anatomic anomalies, had diagnosis of urinary retention, had rUTI, had bladder cancer or pelvic radiation, or had a history of neurological disease/spinal cord injury.	49 cases and 40 controls	Females	Cases: 51 years Controls: 45 years	(Jacobs et al., 2021)
Urinary tract infection (UTI)	Participants with an age of more than 55 years, English-speaking, willing to comply with study procedures (including catheterization for urine sampling), have been prescribed vaginal estrogen and willing to use this for more than 6 weeks prior to sampling.	Participants with instrumentation of urinary tract in prior month, need for chronic intermittent catheterization, history of prior pelvic radiation, active malignancy, breast cancer within previous 5 years or taking any anti-estrogen medication, history of neurologic condition that may affect urinary function, known renal insufficiency with creatinine > 1.3, pregnant or breastfeeding, immunocompromised, immunosuppression, or chronic steroid use, intravaginal pessary use, using only post-coital antibiotic prophylaxis.	64	Females	70.5 years	(Vaughan et al., 2021)
Urgency urinary incontinence (UUI)	Women participants who were prescribed mirabegron for UUI treatment.	Participants with current or suspected UTI, history of rUTI, antibiotic exposure in the past 4 weeks for any reason, immunological deficiency, neurological disease known to affect the lower urinary tract, pelvic malignancy or radiation, untreated symptomatic POP greater than POP-Q stage II or pregnancy.	83	Females	68 years	(Halverson et al., 2022a)
Urgency urinary incontinence (UUI)	Women participants who were prescribed either solifenacin or mirabegron for UUI treatment	Not specified	50 cases and 47 controls	Females	66 years	(Halverson et al., 2022b)
Recurrent urinary tract infection (rUTI)	Symptomatic adult women with an established rUTI diagnosis.	Participants with known anatomic abnormalities of the urogenital tract, neurologic or immunologic disease, a history of bladder malignancy, infection unrelated to previously diagnosed urinary tract disorders.	43	Females	67 years	(Hochstedler et al., 2022)
Various LUTS including UUI, stress UI, UTI, IC/PBS.	Participants previously reported in eight published studies were reanalyzed.	As described in the previous studies.	1004 (UUI = 255, stress UI = 50, UTI = 304, IC/PBS = 49, Controls = 346)	Females	UUI = 65 years, stress UI = 54 years, UTI = 66 years, IC/PBS = 51 years, Controls = 50 years	(Joyce et al., 2022)
Urologic and other surgical procedures	Children undergoing anaesthesia for various procedures.	Participants from Non-primary English-speaking families; with current UTI, antibiotic exposure within the previous 3 months, history of prior urinary tract surgery, immunologic deficiency, neurologic disease affecting the lower urinary tract, or who need to chronically catheterize the bladder.	74	Females (26) and males (48)	2 weeks to 209 months	(Storm et al., 2022)
Conditions without any urinary tract disorders	Healthy menopausal women using vaginal estrogen.	Women with active UTI.	59	Females	Not specified	(Vaughan et al., 2022)

## 2 Urinary tract infection

A number of studies have used EQUC to detect uropathogens from females suffering from urinary tract infections (UTIs). In 2016, Price et al. evaluated the expanded spectrum EQUC using varying volume of urine as well as different cultural and incubation conditions to identify optimum protocol for detecting uropathogens from UTI and non-UTI cohorts (Price et al., 2016). They proposed a streamlined EQUC protocol that detected 84% of uropathogens compared to 34% uropathogens detected by SUC (Price et al., 2016). EQUC also detected higher number of uropathogens (16.15%) from pediatric patients suffering from UTIs compared to SUC (12.80%) (Thapaliya et al., 2020). EQUC was found to detect multidrug-resistant, extensive drug-resistant, and extended-spectrum β-lactamase producing uropathogens as detected by antibiotic susceptibility testing (Thapaliya et al., 2020). While *E. coli* was found to be the most common uropathogen detected by both EQUC and SUC, some other uropathogens such as *Candida albicans*, *Provedencia retegerii*, and *Morganella morganii* grew only on EQUC (Thapaliya et al., 2020). The findings of these studies suggest that the EQUC is capable of providing more useful information to clinicians compared to SUC.

Data from a study have shown that 69% of patients with positive EQUC growth responded to antibiotic treatment (Barnes et al., 2021). Between two types of UTIs detected by EQUC, namely *Escherichia coli*-uropathogen predominant UTIs and non-*E. coli*-uropathogen predominant UTIs, the latter demonstrated better symptom resolution after antibiotic treatment. These results suggest that EQUC may be better utilized to resolve non-*E. coli*-uropathogen predominant UTIs. Another study investigated the association of patient-reported symptoms with urobiome composition in female patients experiencing recurrent UTIs (Burnett et al., 2021). They described five distinct clinical profile groups based on EQUC data collected from 49 participants: odor, cloudiness, and current vaginal estrogen use (no culture result association); frequency, low back pain, incomplete emptying, and vaginal estrogen (significantly increased proportion of *Lactobacillus-*positive cultures); pain/burning, odor, cloudiness, and urgency (high proportions of UTI-associated microbe-positive cultures), frequency, urgency, pain/burning, and current vaginal estrogen use (increased number of no growth cultures) and frequency, urgency, pain/burning, odor, overactive bladder, and sexually active (significantly increased proportion of *Klebsiella*-positive cultures). However, no association of a single urinary symptom was found with specific uropathogens (Burnett et al., 2021). The findings of this study somewhat contradict with the results of another study, which has reported that the presence of pain, but not frequency and urgency of urination is more effective indicators of UTIs in urogynecologic female patients (Dune et al., 2017). The most common uropathogens found in this patient group were *Escherichia coli*, *Enterococuus faeculis*, *Aerococcus urinae* as detected by EQUC. More such studies with greater sample size are warranted to provide useful insights for diagnostic and therapeutic interventions of UTIs.

When detected by SUC, microbial composition has been observed to vary in the same individual due to different urine collection method. The urine collection method has also been observed as a crucial factor while detecting microbes by EQUC method. In a study of 43 women with recurrent UTIs, culture of voided urine as assessed by EQUC yielded high false positive results and the catheterized urine specimens detected microbes with the highest sensitivity (Hochstedler et al., 2022). Additionally, EQUC detected more unique UTI-associated microbes such as *Actinotignum schaalii*, *Candida* species, and *Streptococcus anginosus* and consistently detected more uropathogens from both catheterized and voided urine specimens compared to SUC. These results therefore indicate that EQUC is capable of providing more clinically relevant data.

A recent study with a large number of samples has detected the urinary microbiome present in the catheterized urine specimens collected from female patients with various lower urinary tract symptoms (LUTS) including UTI, urgency urinary incontinence (UUI), interstitial cystitis/painful bladder syndrome [IC/PBS] and stress urinary incontinence (SUI) by EQUC and observed that *Escherichia* was the most prevalent bacterium in UTI patients. (Joyce et al., 2022). Additionally, other bacterial genera including *Lactobacillus*, *Streptococcus*, *Staphylococcus*, *Corynebacterium*, *Actinomyces*, and *Aerococcus* were moderately prevalent in UTI cohort. In another study, different species of *Lactobacillus* were detected in the catheterized urine samples of postmenopausal women with or without UTI as detected by EQUC (Vaughan et al., 2021). It is intriguing that although *Lactobacillus* is generally considered to be a beneficial bacterium, these studies reported to detect *Lactobacillus* species from UTI patients.

## 3 Overreactive bladder and urinary incontinence

The pathophysiology of overreactive bladder (OAB) and urinary incontinence (UI) is not very well-understood. Recent studies using EQUC and NGS have suggested that urobiome may play a role in the pathophysiology of UI. A difference in the urinary microbiota have been observed between OAB/UUI-affected and -unaffected women as detected by EQUC (Hilt et al., 2014; Thomas-White et al., 2016; Thomas-White et al., 2017; Price et al., 2020a). Increased number of microorganisms were detected from the urine samples of the urgency incontinent women compared to the control group (81-85% vs 57-65%) (Thomas-White et al., 2016; Price et al., 2020a). Additionally, relative to control, more types of cultivable bacteria were identified from UUI patients.(Thomas-White et al., 2016) The most frequently isolated bacterial species were found to be *Lactobacillus* spp. and *S. anginonus* in the control group, *Lactobacillus iners*, *S. anginonus* and *S. epidermidis* in SUI patients and *S. anginosus*, *L. gasseri*, *Aerococcus urinae*, and *Gardnerella vaginalis* in UUI patients (Price et al., 2020a). Another study reported that nine genera including *Actinomyces*, *Aerococcus*, *Arthrobacter*, *Corynebacterium*, *Gardnerella*, *Staphylococcus*, *Streptococcus*, *Actinobaculum*, *Aerococcus*, *Arthrobacter and Oligella*) were more frequently isolated from the UUI cohort than from the control group; the last four genera being found only in UUI patients (Pearce et al., 2014). Similar trend was observed in a study conducted by Hilt and colleagues in which while *Lactobacillus*, *Streptococcus*, *Corynebacterium*, *Staphylococcus*, *Actinomyces*, and *Bifidobacterium* spp. were detected from both individuals with and without OAB, *Aerococcus*, *Actinobaculum* and *Athrobacter* were isolated only from OAB patients (Hilt et al., 2014). The previously mentioned study conducted by Joyce and colleagues isolated *S. anginosus, L. gasseri, A. urinae, S. epidermidis, L. iners, Corynebacterium coyleae, Actinomyces neuii, L. jensenii, and Corynebacterium amycolatum* in a more significant number in the UUI patients compared to the healthy control (Joyce et al., 2022). Interestingly, members of the UUI cohort were found to have richer and more abundant urobiome compared to UTI and other cohorts in this study suggesting possible urobiome dysbiosis resulting from overgrowth of bacteria in the UUI patients.

Evaluation of the urobiome profile of patients with UUI before and after treatment have demonstrated longituidinal changes in microbial composition that is associated with symptoms resolution (Thomas-White et al., 2017; Halverson et al., 2022a; Halverson et al., 2022b). In a UUI cohort, the clinical responders to solifenacin, an anticholinergic medication, had a less diverse bacterial community at the baseline, whereas non-responders had a more diverse urobiome (Thomas-White et al., 2016). Additionally, following solifenacin administration, certain microbiota profiles were found to predict the clinically significant response to treatment (Thomas-White et al., 2016). Interestingly, in another UUI cohort treated with mirabegron, a beta-3 agonist approved for treatment of UUI, no difference in the urobiome diversity was observed between the responders and the non-responders at the baseline. However, after 12 weeks of treatment, the urobiome of the responder was found to be significantly richer (Halverson et al., 2022a). In order to evaluate why the microbial characteristics of two cohorts treated by two UUI medication differ, a follow up study was performed to analyze the data obtained from those two studies by using a uniform approach (Halverson et al., 2022b). The re-analysis revealed that alterations in the pre-treatment urobiome occurred in the solifenacin-treated participants only, but not in the mirabegron-treated participants. Additionally, an increased diversity of post-treatment urobiome were found to be associated with treatment response irrespective of medication (Halverson et al., 2022b). Taken together, findings of these studies have indicated that UUI medications affect patients’ microbial profiles differently and further studies are warranted to determine the mechanisms of action of these medications in regards to urobiome.

## 4 Other lower urinary tract symptoms

Apart from UTI and OAB/UI, many patients experience other urinary symptoms involving the bladder, urethra, prostate (in men) and other parts of the lower urinary tract. These symptoms include, but are not limited to, urine hesitancy, intermittent stream, straining, prolonged micturition, feeling of incomplete bladder emptying, nocturia etc (Lepor, 2005). A study examined the association of lower urinary tract microbiota (LUTM) with lower urinary tract symptoms (LUTS) in male patients with and without benign prostate enlargement (BPE) using EQUC and 16S rRNA sequencing and found a positive relationship between LUTM and LUTS (Bajic et al., 2020). LUTM was detected in catheterized urine of 57.1% of men with severe LUTS, 30.0% of men with moderate LUTS, and 22.2% of men with mild LUTS (Bajic et al., 2020). It was revealed that EQUC detected bacteria in 96% of voided urine specimens and 29% of catheterized urine specimens. The bacterial genus *Streptococcus* (mostly *S. anginosus*) and the fungal genus *Candida* were more abundant in catheterized urine of patients with severe LUTS as detected by EQUC (Bajic et al., 2020). These findings might have clinical implications as *S. anginosus* are found to be associated with UUI symptoms in women (Pearce et al., 2014; Joyce et al., 2022) and *Candida* species are associated with patients with symptom flare of urological chronic pelvic pain syndrome (Nickel et al., 2016). All patients with more than 50% relative abundance of *Escherichia* and *Klebsiella* species, two of the most common uropathogens, in their voided urine had moderate-to-severe LUTS (Bajic et al., 2020). When the microbial composition in the samples collected from voided urine, catheterized urine, periurethral swab and transurethral swab of women suffering from pelvic floor symptoms was evaluated, different microbiota was observed in bladder relative to urethral, periurethral and voided urine microbiota (Chen et al., 2020). It has been suggested that this difference in microbiota may be influenced by clinical features such as menopausal status and sexual activity (Chen et al., 2020). Using EQUC protocol, other studies have also demonstrated that the lower urinary tract microbiota has been related to menstruation and vaginal intercourse (Price et al., 2020b).

In a cohort of women with interstitial cystitis/painful bladder syndrome (IC/PBS), *Lactobacillus* and *Streptococcus* were found to be the most common bacteria, detected in 49.0% EQUC positive samples (Jacobs et al., 2021). While *Lactobacillus* was not demonstrated to affect IC/PBS symptom response, *Streptococcus* was found to be associated with less severe symptoms. Additionally, samples collected from most participants with active IC/PBS symptoms did not contain bacteria (Jacobs et al., 2021). Altogether, findings of this study have suggested that bacteria may not impact the symptoms of IC/PBS in the observed cohort.

## 5 Discussion

Ninety percent of urine samples that yielded growth by EQUC protocol have been reported as “no growth” by the SUC protocol (Hilt et al., 2014; Pearce et al., 2014). EQUC is particularly useful in assessing the urine specimens from patients with recurrent UTIs and other urinary symptoms in which the bladder microbes might remain undetected by SUC. Longituidinal studies of microbiota evaluating the symptoms and treatment response of UUI revealed that EQUC is capable of catching microbes that may be used as a predictive biomarker. Therefore, EQUC could potentially be used to detect microbial biomarkers to monitor disease progression, and to develop diagnostic, therapeutic, and prognostic tools (Antunes-Lopes et al., 2020).

With the advent of EQUC, a number of studies have reported that while beneficial commensal bacteria, including *Lactobacillus*, are present in the bladder of healthy females, these bacteria have also been detected in females experiencing urinary symptoms (Hilt et al., 2014; Pearce et al., 2014; Jacobs et al., 2017; Thomas-White et al., 2018; Joyce et al., 2022). It is suggested that the positive and negative contributions of *Lactobacillus* to the bladder health may be determined at the species level and under certain clinical conditions, they can become opportunistic uropathogens (Joyce et al., 2022). It is also possible that the presence of these beneficial bacteria in urinary patients is transient and is linked to symptom responses. A study by Thomas-White and colleagues demonstrated that health-associated commensal bacteria including *L. iners and L. crispatus*, found in both the bladder and vagina of the same individual are functionally highly similar and the authors suggested that these bacteria could provide protection against urinary infections (Thomas-White et al., 2018). Contrarily, a harmful effect of *L. iners* has been suggested in a study by Annelis and colleagues. They proposed that *L. iners* may outcompete Bacillus Calmette-Guerin (BCG), that has been used to manipulate the bladder microenvironment for the treatment of non-muscle invasive bladder cancer, by competing for binding to urothelial fibronectin (Annels et al., 2020). It is intriguing that the same species of *Lactobacillus* may play opposite role under different physiological conditions. Further studies are necessary to investigate the beneficial role of these commensals to evaluate their possible use as biotherapeutics, which will in turn assist to reduce antibiotic-resistant urinary infections. Indeed, the use of this kind of microbiota-mediated treatment must be evaluated considering the health status of the individual patient.

Cumulative studies have suggested that dysbiosis of urobiome influences the microenvironments of the urinary tract and thereby contributing to the onset and progression of the bladder cancer and prostate cancer (Alfano et al., 2016; Annels et al., 2020; Chipollini et al., 2020; Cai et al., 2021; James et al., 2023). Urobiome dysbiosis has also been found to affect the treatment response to the bladder cancer (Annels et al., 2020). Most of these studies have used DNA sequencing methods to detect and characterize the urobiome in cancer patients. A number of anaerobic and other bacteria detected by these methods are not culturable by SUC. EQUC may be utilized to examine whether viable bacteria are required to stimulate the urobiome-mediated tumour-inducing microenvironments. Modulation of the urobiome *via* probiotics, fecal microbiota transplantation and other microbiome-based therapeutics might be useful in preventing and predicting the urinary tract cancers as well as improving treatment response to these cancers.

It is likely that detection of diverse microbial community by EQUC protocol would capture contamination, which is also apparent from the studies that used both voided urine and catharized urine, and found that the latter contained lower load of microbes compared to the former when analyzed by EQUC due to the presence of urethral microbiota in the voided urine (Bajic et al., 2020; Hrbacek et al., 2021; Hochstedler et al., 2022). Vulvovaginal contamination was also observed in clean-catch voided urine (Wolfe et al., 2012). Taken together, these results suggest that catheterized urine specimens detected *via* EQUC would provide clinically relevant information. It is therefore suggested that when analyzed by EQUC, urine samples must be collected by catheter to reduce the chance of contamination (Price et al., 2016).

One limitation of EQUC is its inability to detect fungal species except yeasts. Our understanding of the urinary microbiome is incomplete unless the resident fungal species is considered (Ackerman and Underhill, 2017). The role of the resident fungi and their relationship with the other urinary bacteria warrants future research.

The ability of EQUC to detect a vast array of viable and clinically significant microbes makes it an excellent diagnostic, therapeutic and prognostic tool to study urinary microbiota in health and disease. Besides epidemiological studies, more basic research involving experimental and interventional studies with a larger sample size is warranted to acquire more information regarding the clinical utility of EQUC.

## Author contributions

ND: conceptualization, writing (original draft preparation) and editing. AA: conceptualization, writing and editing. NT: writing. NK: writing. All authors contributed to the article and approved the submitted version.
